# Comprehensive assessment reveals numerous clinical and neurophysiological differences between *MECP2* ‐allelic disorders

**DOI:** 10.1002/acn3.52269

**Published:** 2025-01-21

**Authors:** Davut Pehlivan, Chengjun Huang, Holly K. Harris, Christine Coquery, Aditya Mahat, Mirjana Maletic‐Savatic, Laurence Mignon, Sukru Aras, Daniel G. Glaze, Charles S. Layne, Leonardo Sahelijo, Huda Y. Zoghbi, Matthew J. McGinley, Bernhard Suter

**Affiliations:** ^1^ Section of Pediatric Neurology and Developmental Neuroscience, Department of Pediatrics Baylor College of Medicine Houston Texas 77030 USA; ^2^ Blue Bird Circle Rett Center Texas Children's Hospital Houston Texas 77030 USA; ^3^ Texas Children's Hospital Houston Texas 77030 USA; ^4^ Jan and Dan Duncan Neurological Research Institute at Texas Children's Hospital Houston Texas 77030 USA; ^5^ Section of Developmental Pediatrics, Department of Pediatrics Baylor College of Medicine Houston Texas 77054 USA; ^6^ Ionis Pharmaceuticals Carlsbad California 92010 USA; ^7^ Department of Health and Human Performance University of Houston Houston Texas USA; ^8^ Center for Neuromotor and Biomechanics Research University of Houston Houston Texas USA; ^9^ Center for NeuroEngineering and Cognitive Science University of Houston Houston Texas USA; ^10^ Department of Neuroscience Baylor College of Medicine Houston Texas 77030 USA; ^11^ Present address: University of Health and Rehabilitation Sciences Qingdao City Shandong Province China

## Abstract

**Objective:**

Rett syndrome (RTT) and *MECP2* duplication syndrome (MDS) result from under‐ and overexpression of *MECP2*, respectively. Preclinical studies using genetic‐based treatment showed robust phenotype recovery for both MDS and RTT. However, there is a risk of converting MDS to RTT, or vice versa, if accurate MeCP2 levels are not achieved. The aim of this study was to identify biomarkers distinguishing RTT from MDS.

**Materials and Methods:**

We prospectively enrolled 11 MDS and 6 male RTT like (MRL) individuals for a panel of clinical and neurophysiological assessments over two visits, 8–10 months apart.

**Results:**

We identified numerous clinical and physiological features as promising biomarkers. MRL individuals exhibited large amplitude whole body tremor, midline stereotypies (vs. hand flapping at sides in MDS), earlier neuromotor regression, and earlier onset but less commonly refractory epilepsy. In the neurophysiological domain, we observed several marked differences in sleep physiology between MDS/MRL and typically developing (TD) individuals including reduced sleeping time, increased delta power during rapid eye movement (REM) sleep, decreased occipital alpha and increased brain‐wide delta power during wakefulness, and reduced spindle density and duration. MRL individuals also had much lower delta power during NREM 2 and 3 stages than the TD group. We found differences in spindle duration in the temporal lobes and spindle amplitude in the frontal lobes between MDS and MRL.

**Discussion:**

Our study revealed distinct clinical features of MDS and MRL that can be monitored during a clinical trial and may serve as target engagement, disease progression, or safety biomarkers for interventional studies.

## Introduction

The MeCP2 protein binds to methylated cytosines, and regulates expression of thousands of genes.^1^, ^2^ Loss of function or deletion mutations of *MECP2* cause Rett syndrome (RTT, MIM# 312750),^3^ a severe neurodevelopmental disorder (NDD) characterized by developmental delay/intellectual disability (DD/ID), dysautonomia, epilepsy, gastrointestinal problems, sleep disturbances, and hand stereotypies.^4^ However, there are males with loss of function or hypomorphic alleles who survive into infancy or childhood and demonstrate RTT‐like features.^5^, ^6^, ^7^, ^8^, ^9^, ^10^, ^11^, ^12^, ^13^, ^14^

Conversely, extra copies of *MECP2*, including duplications and triplications, are known to cause *MECP2* Duplication Syndrome (MDS, MIM# 300260). Similar to RTT, MDS is a severe NDD with core clinical features that include congenital hypotonia, motor difficulties, DD/ID, epilepsy, gastrointestinal problems, and recurrent respiratory infections usually observed in males.^15^, ^16^

Genetic‐based treatments are revolutionizing medicine, especially for neurological disorders.^17^, ^18^, ^19^ Currently, there is no approved genetic based treatment for MDS or RTT however, a non‐genetic‐based treatment, trofinetide, has recently been approved for RTT.^20^ Preclinical studies in mice have shown rescue of the RTT phenotypes using gene therapy^21^, ^22^ and MDS phenotypes using antisense oligonucleotide treatment.^23^, ^24^ Importantly, there is a risk of phenoconversion when an appropriate balance in MeCP2 is not achieved. Thus, for targeting dosage‐sensitive genes like *MECP2*, accurate dosing and objective biomarkers to assess response to therapy are critically important, particularly when there is significant overlap in the clinical presentation.^25^, ^26^, ^27^, ^28^ Over the last decade, there has been a notable surge in biomarker research led by collaborative efforts from the FDA and NIH to establish shared definitions and develop the BEST (Biomarkers, EndpointS, and other Tools) Resource.^29^ As there are challenges with measuring MeCP2 directly (i.e., MeCP2 is not a soluble protein and functions in the nucleus thus, measuring plasma/CSF levels may not represent MeCP2 levels in the brain), surrogate, pharmacodynamic biomarkers (i.e., those that change in response to therapy) will be of great value for future genetic‐based trials.

Currently, there are not clearly defined clinical, neurophysiological, or molecular biomarkers that can be used to monitor for changes in *MECP2* in either MDS or RTT. Therefore, we (1) sought to identify clinical and neurophysiological features with sufficient sensitivity and specificity to differentiate between MDS, RTT, and typical populations, and (2) assess the stability and reliability of these features over time, with the overarching goal of using them to determine the response to target engagement and to monitor safety in future interventional trials.

## Materials and Methods

### Study design and participants

This prospective study enrolled patients with MDS and male individuals who have loss of function mutation in *MECP2* (termed male Rett‐like, MRL) and performed a battery of clinical and neurophysiological assessments. We purposefully compared male MDS and MRL individuals to prevent the second X‐chromosome's biological bias. Neurophysiological data from neurotypical controls was obtained from retrospective analysis of polysomnography studies. The study was approved by the Baylor College of Medicine Institutional Review Board (Protocol number: H‐46532).

### Clinical assessments

#### MECP2‐related disorders specific history and physical examination

We developed a MECP2‐related disorders history and physical examination form based on the literature and our center's clinical experience. This clinical assessment form covered clinical features encompassing, MDS and RTT (including MRL) clinical features, to allow comparison between overlapping clinical features as well as tracking of features distinct to each condition.

#### Neurodevelopmental assessment

We directly assessed neurodevelopmental skills using the Capute Scales or CAT/CLAMS (Cognitive Adaptive Test/Clinical Linguistic and Auditory Milestone Scales).^30^ We also obtained parent report of language, daily living, social, and motor skills via a standardized measure, the Vineland Adaptive Behavior Scales, Third Edition Comprehensive Interview Form (Vineland‐3).

#### Gait assessment

Ambulatory patients underwent gait study via overground walking on a standardized instrumented walkway. The overground walking consists of walking over a GAITRite^®^ instrumented mat 61 cm wide × 4.27 m long (GAITRite^®^). The GAITRite contains embedded pressure activated sensors which the customized software translates into spatiotemporal gait measures. During testing, if an individual was unable to complete a walkway pass, the trial was stopped and repeated until four complete trials were obtained.

Kinematic and force data were used in combination to identify heel strike and toe off as described previously by our group.^31^ Verbal encouragement and occasional physical assistance, such as a touch on the shoulder, was provided as appropriate.

#### Actigraphy

We measured patterns of physical activity and sleep using an actigraph, PhilipsActiwatch (Philips Respironics, Bend, Oregon), placed on the dominant wrist. The actigraph collected data in 30‐s epochs, day and night, for seven consecutive days, including at least three consecutive days in the home environment. The first night of actigraphy overlapped with the polysomnography (PSG) study, allowing accurate correlations with neurophysiology data.

Data were split into three intervals: (1) noon–sleep onset, (2) sleep, and (3) wake–noon intervals. To standardize activity values, activity measures were summed and divided by duration of the measured non‐sleep interval.

#### Skin conductance and thermal imaging

We quantified autonomic thermal dysregulation of the extremities (cool hands and feet) using a portable infrared thermal camera (FLIR T540 Thermal IR Imaging) to measure skin temperature at defined landmarks on the hands and feet following at least 5 min of rest. The infrared thermal camera was placed orthogonal to the body part (hands and feet) approximately 50 cm from the skin's surface. Individuals were not involved in vigorous physical activity prior to assessments.

### Neurophysiologic assessments

#### PSG study

Electroencephalogram (EEG) signals were acquired using the research standard 10–20 international system with a total of 13 scalp electrodes (Fp1/2, C3/4, O1/2, F7/8, T3/4, Fz, Cz, Oz). We used 13 scalp electrodes to increase spatial resolution over routine clinical PSG. The signal for each electrode was referenced to an ear mastoid site. The EEG sampling rate was 500 Hz. Raw EEG data were filtered with a zero‐phase band‐pass filter (20‐order Butterworth, 0.1–30 Hz) and down‐sampled to 200 Hz. See “Supplemental Materials and Methods” for further details.

#### Visual evoked potentials

Visual evoked potentials (VEPs) were assessed using a contrast reversing black/white checkerboard. Monocular testing was performed since binocular stimulation may mask a unilateral visual conduction abnormality. The patient was seated at a fixed distance (60 cm) from the screen and was encouraged to focus on the center of the screen. Recording electrodes were placed at vertex (Cz), mid‐occipital; (Oz), earlobe (A1), and forehead (Fz). The checkerboard pattern was reversed (black to white to black) at a rate of 2 per second. The resulting N1, P1, and N2 were defined and identified as in LeBlanc et al., by a neurophysiologist highly experienced in reading VEP.^32^

### Statistical analyses

We used frequency and percentages for categorical variables and clinical features, and mean and standard deviation for continuous variables. We executed chi‐squared test while comparing categorical variables, and Mann–Whitney *U*‐test for continuous variables. All statistical analyses were conducted using SPSS version 29.0 and R Studio program. *P*‐value <0.05 was considered statistically significant.

For the sleep and polysomnography study, all statistical analyses were performed by built‐in MATLAB functions (version 2022a). We used Wilcoxon signed rank test for differences in the percentage of time spent in each PSG stage between the two visits for each MDS patient. Because some differences were observed, we did not average those two visits together, and instead included both visits as independent, in subsequent analyses. We used Kruskal–Wallis ANOVA test to compare the difference in wake and sleep stages percentages between typically developing (TD), MDS, and MRL groups, with Tukey's post hoc for multiple comparison correction. See “Supplemental Materials and Methods” section for further details.

We also established a scoring table for clinical and neurophysiological data to evaluate any differentiating scores between TD, MDS, and MRL. The rationales for developing this scoring system were to (1) guide clinicians for dose adjustments with potential treatments (e.g., if positive clinical score converts to negative score, it can alert clinician/researcher for over treatment) and (2) determine specific neurophysiological cut off values that can differentiate MDS, MRL, and TD for different sleep phases (REM, NREM1‐3). For the clinical features that showed differences between MDS and MRL (i.e., tremor, midline hand stereotypy, abnormal breathing pattern, absence of refractory seizures, and absence of respiratory infection), a value of “−1” was used for features that are present in MRL group, “+1” for features that are present in MDS group, and “0” for individuals who are not carrying the feature. For the neurophysiological data, we examined awake, NREM1, NREM2‐3, and REM data between TD, MDS, and MRL. We executed receiver operating characteristic (ROC) analysis to determine differentiating cutoff values between TD, MDS, and MRL. Identified cutoff values were assessed by confusion matrix and its performance metrices for grouping.

## Results

### Baseline characteristics

**Figure 1:**
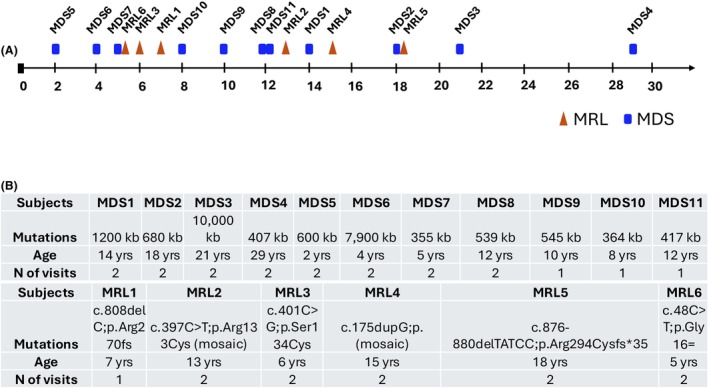
General characteristics of *MECP2* duplication syndrome (MDS) and male Rett like (MRL) individuals. (A) Age distribution of MDS (blue rectangle) and MRL (orange triangle) individuals. (B) Mutations details including size of duplication in MDS individuals and simple nucleotide variant details in MRL individuals, age at enrollment, and number (*N*) of visits per individual.

A total of 17 individuals (11 MDS and 6 MRL) enrolled in the biomarker study (Table S1). The study aimed for all participants to undergo two visits 8–10 months apart for longitudinal assessment of the findings from the Visit #1. However, due to COVID restrictions, inclusion in each assessment varied (Table S2). Participant age ranged from 2 to 29 years (with a mean age of 12.3 years old) for the MDS individuals and 5–18 years (with a mean age of 10.7 years old) for the MRL individuals (Fig. 1A). Duplication size for MDS individuals ranged from 355 to 10,000 kb and each MRL participant had a different variant (Fig. 1B).

### Clinical assessments

Cross‐sectional results from the comprehensive clinical evaluation revealed five distinctive features between MDS and MRL including tremor, stereotypy characteristic, frequent respiratory infection, age of neuromotor regression, and age of seizure onset (Table 1). Of note, high amplitude resting and action tremors including extremities and torso were present in all six MRL individuals that were absent in all MDS individuals (*P* < 0.0001). Stereotypies were observed in both MDS (10/11, 90%) and MRL (5/6, 83%), their characteristics were different. MDS individuals exhibit hand flapping and body rocking stereotypies similar to those seen in idiopathic autism. MRL individuals exhibit midline hand wringing/clasping type stereotypies classic for RTT. Frequent respiratory infections were almost universal in MDS (10/11, 90%) whereas they were observed in only 1 out of 6 (16%) MRL individuals (*P* = 0.005). Eight out of 10 individuals reporting frequent respiratory infections had frequent pneumonias and the other two individuals had frequent upper respiratory infections. A single MDS individual who at the time of assessment was not experiencing frequent infections had a history of frequent upper respiratory infections that he outgrew. The mean age of neuromotor regression in MRL was 1.5 years of age. In contrast, MDS individuals regressed in the setting of worsening of epilepsy at a mean age of 11.5 years (one outlier in each group). While epilepsy frequency was similar in both groups (MDS: 8/11 [72%]; MRL: 4/6 [66%], *P* = 1.0), MDS individuals were more refractory to treatment (MDS: 6/8 [75%]), MRL: (1/4 [25%], *P* = 0.22). Average age of seizure onset significantly differed (MDS: 10.5 [4–17] years; MRL: 32 [20–29, 31–61] months, *P* = 0.03).

**Table 1:** Summary of history and physical examination findings.

Clinical feature	MDS (*N* = 11)	MRL (*N* = 6)	*P*‐value
*Tremor (large amplitude, whole body)	0/11 (0%)	6/6 (100%)	0.000002
*Stereotypies characteristics	At sides, not interfering with hand function	Midline, interferes with hand function	NA
Stereotypy frequency and type	10/11 (90%), hand flapping at side and body rocking	5/6 (83%), hand wringing/clasping	1.0
Poor eye contact	5/11 (45%)	3/6 (50%)	1.0
*Frequent respiratory infections	10/11 (90%)	1/6 (16%)	0.005
*Regression age, reason	11.5 years, worsening of epilepsy	1.5 years, no external reason	0.0035
Epilepsy frequency, refractory status, *age of onset	8/11 (72%) had epilepsy, 6/8 (75%) had refractory seizures, age of onset 10.5 years	4/6 (66%) had epilepsy, 1/4 (25%) had refractory seizures, age of onset 32 months (2.6 years)	1.0 for epilepsy frequency, 0.22 for refractory status, 0.03 for age of seizure onset
Constipation	10/11 (90%)	5/6 (83%)	1.0
Feeding‐chewing difficulty/G‐tube requirement	11/11 (100%) / 5/11 (45%)	5/6 (83%) / 3/5 (60%)	0.35/1.0
GERD	7/11 (63%)	4/6 (66%)	1.0
Dysautonomia	10/11 (90%)	5/6 (83%)	1.0
Dysautonomia severity	++ (drooling, cool hands and feet)	+++ (drooling, cool hands and feet, abnormal breathing pattern)	NA
Insomnia	6/11 (54%)	4/6 (66%)	1.0
Sleep apnea	8/8 mild OSA (100%), 6/8 were identified during sleep study and families were not aware of it)	5/5 (100%), 4/5 has moderate to severe OSA	1.0
Genitourinary abnormality	4/11 (36%), all 4 had cryptorchidism including 2/4 microphallus	2/6 (33%), both of them had cryptorchidism only	1.0
Bruxism	11/11 (100%)	5/6 (83%)	0.35
High pain tolerance	11/11 (100%)	6/6 (100%)	1.0
Self‐mutilation	2/11 (18%)	3/6 (50%)	0.28
Overactive or overly passive	6/11 (54%)	4/6 (66%)	1.0

*Note:* Statistically different clinical features are denoted with an “*”.

GERD, gastroesophageal reflux disease; MDS, MECP2 duplication syndrome; MRL, male Rett‐like; NA, not applicable.

Remaining clinical assessments, neurodevelopmental, gait, actigraphy, and infrared thermal assessments are provided in the Notes S1.

### Polysomnography/EEG

Sleep disturbances are highly prevalent in NDDs including RTT,^33^, ^34^, ^35^ and has been reported anecdotally in MDS.^36^, ^37^ We analyzed sleep studies from 11 MDS and 6 MRL individuals during Visit 1, and 6 MDS and 5 MRL individuals during Visit 2 (Table S2). MRL individuals' PSG data were not analyzable for Visit 2, because there were insufficient data during sleep due to a shortened duration of the visit due to COVID restrictions, combined with the low percentage of time asleep for this population. Thus, we excluded Visit 2 for MRL individuals from further analysis. We also retrospectively analyzed 150 age‐matched sleep studies of TD individuals who underwent sleep study for various reasons at Texas Children's Hospital (7.9 ± 4.6 years, 68 females) as a control group for comparison.

#### MDS individuals have decreased time asleep and reduced time in REM

To investigate whether sleep architecture is disrupted in MDS and/or MRL groups, we compared the percentage of time spent in each manually scored stage (Wake, NREM1, NREM2/3, and REM) during each night's sleep study. Substantial differences were observed in time spent awake and in the various sleep stages. The MDS individuals had a significantly larger percentage of the time awake and, while asleep, spent significantly more time in the combined NREM2‐3 stage, and less in the REM stage, compared to the TD group; however, no differences were noted between MDS and MRL groups. See Notes S1 and Figure S1 for details.

#### Altered spindle characteristics distinguish MDS from MRL

A sleep spindle is a burst of EEG oscillations in the frequency range of 12–15 Hz, which is observed during NREM stages in neurotypical subjects,^38^ and plays an important functional role in sleep‐dependent synaptic plasticity and memory consolidation.^39^ Spindle characteristics have been used as markers of brain development in infants and individuals with NDD.^39^ To investigate whether spindle characteristics are altered, and thus could be used as biomarkers for MDS or MRL individuals, we used the Luna spindle detector algorithm (see Methods) to detect spindles from NREM2‐3 stages. The spindle density, spindle duration, and normalized spindle amplitude were calculated.

**Figure 2:**
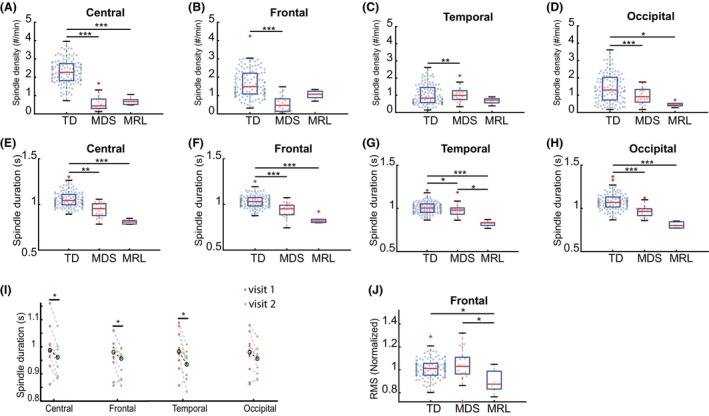
Altered sleep spindle properties in MDS and MRL. (A–D) Spindle density (spindles per minute) averaged across leads within each of the indicated brain regions. The TD group had a significantly higher spindle density than both the MDS and MRL group in the central and occipital region. The TD group had a significantly higher spindle density than the MDS group in the frontal and temporal region. (E–H) Spindle duration (s) averaged across leads within each of the indicated brain regions. The TD group had a significantly longer spindle duration than the MDS, and MRL group in the central, frontal, and occipital regions. The spindle duration of the MRL group was significantly shorter than the MDS group at the temporal region. (I) Comparison of spindle duration between the first and second visit for MDS individuals. The spindle duration during the first visit (*N* = 9) was significantly longer than the second visit at the central, frontal, and temporal regions. (J) The root mean squared (RMS) amplitude after normalizing to the mean across all leads and time points. The MDS group had a significantly larger normalized spindle amplitude than the TD, and MRL group in the frontal region. The black circle represents the median value for each condition. Comparison of spindle density, duration and amplitude at each region between groups was performed by the Kruskal–Wallis test followed by Tukey's post hoc multiple comparison test. (*P < 0.05; **P < 0.01; ***P < 0.001). Box plots are similar to Figure 1. Red “+” indicates values outside the quantile range. Results are from 19 MDS, 6 MRL, and 150 TD visits.

Spindle density was compared between MDS, MRL, and TD groups. A Kruskal–Wallis test showed that there was a significant difference of spindle density at the group level for multiple brain regions (central, chi square = 49.66, *P* < 0.001; frontal, chi square = 29.67, *P* < 0.001; temporal, chi square = 13.09, *P* < 0.01; occipital, chi square = 33.45, *P* < 0.001). The TD group had a significantly higher spindle density than the MDS group in all regions (Fig. 2A–D), and a significantly higher spindle density than the MRL group at the central (Fig. 2A) and occipital (Fig. 2D) regions, but not at the frontal (*P* = 0.31) and temporal (*P* = 0.46) regions. No significant differences in spindle density between MDS and MRL were found across the four regions (central, *P* = 0.77; frontal, *P* = 0.93; temporal, *P* = 0.75; occipital, *P* = 0.98). For the MDS individuals with two visits, the spindle density during the first visit was not significantly different from the second visit across the four regions (frontal: *P* = 0.65; central: *P* = 0.49; occipital: *P* = 1.00; temporal: *P* = 0.91).

Spindle duration was then compared between the groups. A Kruskal–Wallis test showed that there was a significant difference of spindle duration at the group level across four regions (central, chi square = 29.42, *P* < 0.001; frontal, chi square = 31.07, *P* < 0.001; temporal, chi square = 22.31, *P* < 0.001; occipital, chi square = 39.21, *P* < 0.001). The TD group had a significantly longer spindle duration at all regions than both the MDS and MRL group (Fig. 2E–H). The spindle duration of the MRL group was significantly shorter than the MDS group at the temporal region (Fig. 2G), but was not different than the MDS at central (*P* = 0.10), frontal (*P* = 0.17), and occipital (*P* = 0.26) regions. The spindle duration of the MDS individuals' first visit was significantly longer than the second visit at the central, frontal, and temporal regions (Fig. 2I), and was marginally significant for the occipital region (*P* = 0.10).

We last characterized the spindle amplitude. A Kruskal–Wallis test showed that there was a significant difference of spindle amplitude at the group level for the frontal region (chi square = 6.36, *P* < 0.05), but no significant differences were found at the other three regions (central, *P* = 0.06; occipital, *P* = 0.21; temporal, *P* = 0.06). Both the TD group and MDS group had a significantly larger normalized spindle amplitude than the MRL group in the frontal region (Fig. 2J). No significant difference was found between TD and MDS group in the normalized spindle amplitude (*P* = 0.84). No significant differences of normalized spindle amplitude were found between the two visits of MDS individuals across the four regions (frontal, *P* = 0.30, central, *P* = 0.49, occipital, *P* = 0.30, temporal, *P* = 0.36).

Taken together, our analyses of spindles shows that spindle density and duration are altered significantly in both MDS and MRL individuals compared to TD and there is a difference for spindle duration in the temporal lobes and spindle amplitude in the frontal lobes between MDS and MRL.

#### Altered power spectra in multiple sleep stages in MDS

Spectral power in particular frequency bands are the defining characteristics of the major sleep stages.^38^ Therefore, we next used temporally resolved spectral analyses to assess changes of sleep EEG power in MDS and MRL. We used a multitaper approach (see Methods) to calculate power spectrum density (PSD) of the sleep EEG signals from each electrode, in 30 s epochs. We then calculated the spectral power in each of six frequency bands (see Methods). We first compared the power in each frequency band between the first and second visit of the MDS individuals. We found no significant differences between visits for any of the four regions, frequency bands, or regions (*P* values ranged from 0.06 to 1.0). The *P*‐value for the delta power at the center and frontal region in MDS individuals, between visits was 0.055, indicating a marginally significant result. We then compared our power metrics for all six frequency bands, across the three subject groups (MDS, MRL, and TD) and four sleep stages (wake, NREM1, NREM2‐3, and REM).

**Figure 3:**
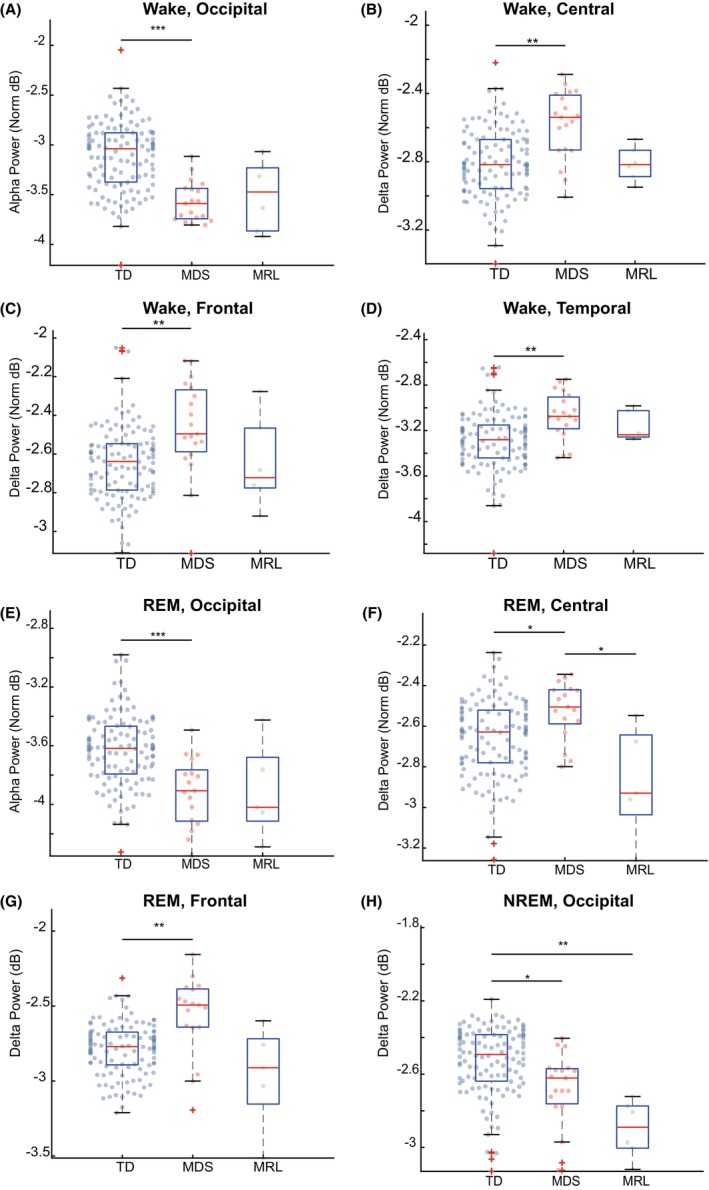
Altered alpha and delta power in MDS and MRL. (A) Power in the alpha band (8–10 Hz) for the occipital lobe recording sites, normalized to the average power from −0 to 30 Hz across all leads and time points. This power was decreased significantly in the MDS group. (B–D) Same as in (A), but for delta band (2–4 Hz) in the indicated brain areas. The MDS group had significantly higher delta power than the TD group at the frontal, central and temporal regions during wake stage. (E) The alpha band power of MDS group was decreased significantly at the occipital region compared to TD group during REM stage. (F) The delta band power of MDS group was significantly larger than both TD, and MRL group at the central region during REM stage. (G) The delta band power of MDS group was increased significantly at the frontal region compared to TD group during REM stage. (H) The delta band power of MRL group was decreased significantly compared to TD group at the occipital region during NREM stage. Comparison of the normalized PSD at each region between groups was performed by the Kruskal–Wallis test followed by Tukey's post hoc multiple comparison test. (*P < 0.05; **P < 0.01; ***P < 0.001 after multiple correction). Box plots are similar to Figure 1. Red “+” indicates values outside the quantile range. Results are from 19 MDS, 6 MRL, and 150 TD visits.

We first focused on the wake stage. The wake stage is typically characterized by low overall signal amplitude (compared to NREM, particularly in the delta band) and high alpha power in the occipital lobe, which is associated with offline visual processing.^40^ Significant effects were observed for delta and alpha bands in some brain areas. In particular, a Kruskal–Wallis test showed group‐level significance for delta power in all four regions (central, chi square = 16.12, *P* < 0.001; frontal, chi square = 12.20, *P* < 0.001; temporal, chi square = 14.23, *P* < 0.001; occipital, chi square = 8.23, *P* < 0.05). The post hoc pairwise comparisons found that delta power of the MDS group was significantly larger than the TD group for the central (Fig. 3B), frontal (Fig. 3C), and temporal (Fig. 3D) but not occipital (*P* = 0.24, Fig. S2A) regions. No significant differences in delta power were found between TD and MRL groups for central (*P* = 1.00, Fig. 3B), frontal (*P* = 1.00, Fig. 3C), temporal (*P* = 1.00, Fig. 3D), and occipital (*P* = 1.00, Fig. S2A) regions, or between MDS and MRL groups for central (*P* = 0.53, Fig. 3B), frontal (*P* = 0.80, Fig. 3C), temporal (*P* = 1.00, Fig. 3D), and occipital (*P* = 0.11, Fig. S2A) regions.

For the alpha band in the wake stage, significant difference at the group level was seen for central (chi square = 6.98, *P* < 0.05) and occipital (chi square = 31.56, *P* < 0.001) regions, but not frontal (*P* = 0.92, Fig. S2H), and temporal (*P* = 0.50, Fig. S2I) regions. Post hoc comparisons found that the alpha power of MDS individuals was significantly lower than TD, for the occipital region (Fig. 3A), but not different for the central region (*P* = 0.15, Fig. S2G). No significant differences in alpha power were found between TD and MRL groups at the central (*P* = 1.00, Fig. S2G) or occipital (*P* = 0.20, Fig. S2A) regions, or between MDS and MRL groups at the central (*P* = 1.00, Fig. S2G) and occipital (*P* = 1.00, Fig. 3A) regions. Therefore, although there is a group level significant difference in alpha power for the central region, there is no significant difference between any two groups after the multiple comparison correction.

Taken together, power spectral patterns for the wake stage are significantly altered in MDS individuals. In particular, MDS individuals have increased delta power across all regions, and decreased alpha power in the occipital region. In MRL individuals, power spectral patterns for the wake stage are not significantly altered from TD.

We next assessed the power in our six frequency bands across brain areas during pooled NREM2 and three stages. The NREM2‐3 stages in neurotypical individuals are characterized by high delta power.^38^, ^41^ A Kruskal–Wallis test showed that there was group‐level significant for the delta band in the central (chi square = 8.90, *P* < 0.05) and occipital (chi square = 22.63, *P* < 0.001) regions, but not frontal (*P* = 0.18, Fig. S2E), or temporal (*P* = 0.56, Fig. S2F). The post hoc pairwise comparison found that the MRL individuals had significantly lower delta power than the TD group in the occipital region (Fig. S2H), but similar to TD group at the central region (*P* = 0.16, Fig. S2D). No significant differences were found between TD and MDS groups at the central region (*P* = 1.00, Fig. S2D) and there was a significant difference between TD and MDS group at the occipital region (Fig. 3H). No significant differences were found between MDS and MRL groups for the central (*P* = 1.00, Fig. S2D) and occipital (*P* = 1.00, Fig. 3H) regions.

Taken together, power spectral analysis for the NREM2‐3 stages show reduced delta power in MRL and MDS, suggesting altered slow oscillation activity in the two populations.

The final sleep stage for which we analyzed power spectral patterns is the REM stage. REM in neurotypical populations exhibits low muscle tone (low EMG intensity) and similar spectral features to the wake stage, with the exception of occipital alpha, which is transient and lower in power between 9.5 and 10 Hz in REM.^41^, ^42^ In our data for REM, a Kruskal–Wallis test showed significantly altered delta power in all regions (central, chi square = 13.09, *P* < 0.01; frontal, chi square = 16.52, *P* < 0.001; temporal, chi square = 8.69, *P* < 0.05; occipital, chi square = 7.24, *P* < 0.05) at the group level. Mirroring the effects in the wake stage, in REM stage the delta power was altered in the MDS group compared to the TD group. The delta power, which is usually not strong in REM stage in TD populations, was stronger in the MDS group than the TD group for the central region (Fig. 3F) and frontal region (Fig. 3G).

The MDS group also had higher delta power than the MRL group in the central region (Fig. 3F). Although the Kruskal–Wallis test also showed group‐level significant differences of delta power for the temporal and occipital regions, the pairwise comparisons were not significant after correcting for multiple comparisons (occipital, TD vs. MDS, *P* = 1.00, MDS vs. MRL, *P* = 0.11, TD vs. MRL, *P* = 0.17, Fig. S2B; temporal, TD vs. MDS, *P* = 0.07, MDS vs. MRL, *P* = 0.88, TD vs. MRL, *P* = 1.00, Fig. S2C). Group‐level significant difference in the alpha power during REM stage was found for the occipital (chi square = 19.38, *P* < 0.001), but not central (*P* = 0.73, Fig. S2J), frontal (*P* = 0.55, Fig. S2K), or temporal (*P* = 0.85, Fig. S2L) regions. The alpha power at the occipital region was significantly decreased in the MDS group compared to TD group (Fig. 3E). No significant differences in alpha power were found between TD and MRL groups (*P* = 0.59, Fig. 3E), or between MDS and MRL groups (*P* = 1.00, Fig. 3E) for the occipital region.

Taken together, power spectral patterns for the REM stage are significantly altered in MDS individuals. In particular, MDS individuals have increased delta power for central and frontal regions and decreased alpha power in the occipital region. In MRL individuals, power spectral patterns for the REM stage are not significantly altered.

Our overall spectral analysis results show that, compared to TD individuals, MDS individuals have altered delta and alpha power in wake and REM stages, and MRL individuals have altered delta power in NREM2/3 stages.

The remaining electrophysiological studies, including VEP assessments, are provided in the Notes S1.

### Determining scoring values to differentiate between TD, MDS and MRL groups

We developed a scoring system that can differentiate TD, MDS and MRL. There were five clinical features that differ between these three groups. Based on −1/0/+1 points for each clinical feature, all MDS individuals had positive values, MRL individuals had negative values and TD individuals were “0” with a sensitivity and specificity of 100%.

For the sleep study, awake values were differentiating statistically significant (*P* < 0.001) MRL from TD based on cutoff value of 26.65 according to ROC analysis (Table S3, area under curve [AUC] = 0.991). Awake values were not striking enough for differentiating MDS from TD despite statistically significance (*P* < 0.001, AUC = 0.848), thus we did not determine a cutoff value. Thus, we developed a formula [(Awake+NREM1) × 10/REM] based on ROC analysis that has significant impact on the differentiation between TD, MDS (cutoff = 13.5, *P* < 0.001, AUC = 0.927) and MRL (cutoff = 21.1, *P* < 0.001, AUC = 0.993) (Table S3).

## Discussion

We conducted a prospective, non‐interventional clinical study to identify clinical and electrophysiologic differences between male *MECP2* overexpressors (MDS) and underexpressors (MRL). The purpose was to identify biomarkers/outcome measures that might indicate over engagement of the target. Our study highlighted key clinical and neurophysiological differences for these allelic *MECP2*‐related disorders.

### Comprehensive clinical studies

There are several historical and physical examination finding differences between MDS and MRL. Tremor and stereotypies can differentiate MDS and MRL; infection is an important differentiating factor but difficult to assess clinically in the short term; age of neuromotor regression, age of epilepsy onset, severity of epilepsy, neurodevelopmental levels, dysautonomia (particularly breathing abnormalities), and actigraphy are other differentiating features that can be used as long‐term outcome measures/biomarkers in interventional studies.

Large amplitude whole‐body tremor was seen in the great majority of MRL individuals and was not observed in MDS individuals. Moreover, additional MRL individuals who did not participate in this study but were clinically evaluated at our center had whole body tremor. Thus, tremor should be considered as one of the key clinical features differentiating MDS and MRL. Tremor has not been considered a defining feature of RTT. Importantly, there are several MRL case reports and case series and tremor (as truncal titubation in one of them) was reported only in three families.^7^, ^10^, ^14^ Among them, the largest cohort was composed of 30 MRL individuals and reported the clinical and molecular details.^5^ However, the authors focused on RTT main and supportive criteria, leaving out tremor assessment and thus it was not reported in their series.

Autism spectrum disorder has been reported in both MDS and RTT. Peters et al. compared MDS individuals with age‐matched individuals with idiopathic autism and showed that social impairment and repetitive behaviors are similar in both groups.^43^ Stereotypies in MDS are mainly hand flapping at the sides and body rocking, which are also common stereotypies in idiopathic autism. On the other hand, stereotypies in MRL are mainly midline hand wringing and tapping/clasping which is similar to classic RTT individuals. Female RTT individuals have a broader spectrum of stereotypies, and interestingly hand mouthing and clapping/tapping was more common than wringing stereotypy in children.^44^ Additionally, midline hand stereotypy has been reported in at least one MDS cohort study, but the incidence rate is not known.^45^ Our center has also evaluated one child with MDS who had midline hand stereotypy; this child did not participate in the current study. However, hand flapping and body rocking at sides were still predominant stereotypies, and midline hand stereotypy was rarely observed in our subject. Thus, it is important to characterize baseline stereotypy semiology in MDS as individuals with MDS may have midline stereotypy as well.

Dysautonomia is one of the defining features of classic female RTT individuals. Similarly, dysautonomia was almost a global finding for both MRL and MDS. However, severity of dysautonomia in the MRL group was similar to female RTT individuals and included breath holding and hyperventilation symptoms in addition to drooling and cold/cool‐discolored extremities. Thus, dysautonomia is more severe in MRL individuals compared to MDS individuals. Similarly, Neul et al. identified periodic breathing as a common feature in MRL individuals in their large MRL cohort.^5^

There are also certain features that differ between MDS and MRL that require prolonged time to emerge, and thus probably are not useful outcome measures in clinical trials. These include age of neuromotor regression, epilepsy age of onset, and recurrent infections. Recurrent respiratory infections are one of the defining features of MDS in OMIM. In our cohort, 10 out of 11 MDS individuals had recurrent respiratory infections while the presence in MRL individuals was much rarer. Based on our clinical experience, at least female RTT individuals are prone to frequent respiratory infections especially in individuals with poor seizure control and terminal disease. The lack of frequent respiratory infections in MRL individuals may be due to caregivers being extra cautious. We previously showed that recurrent infections are severe and cause hospitalizations in more than two out of three of MDS individuals (1/3 of these are intensive care unit admissions), thus causing significant burden to caregivers of individuals with MDS.^46^ However, as respiratory infections occur rarely (at most a few times a year), it would be difficult to use as an outcome measure in MDS in the short term. Age of onset for epilepsy and for neuromotor regression also differ significantly between MDS and MRL. The average age of onset for seizure was 10.5 years versus 32 months in MDS and MRL, respectively. Similarly, the average age of neuromotor regression for MDS and MRL were 11.5 and 1.5 years, respectively, and neuromotor regression in MDS was attributed to seizure onset/worsening, while no external contributors identified in the MRL group. Additionally, while the majority of epilepsy in MDS individuals is refractory, severity of epilepsy in MRL varies (75% vs. 25%, *P* = 0.22). Lack of statistical significance despite threefold difference is probably related to low sample size. Additionally, the refractory status in MDS and MRL are similar to published literature (~80% of MDS individuals and one third in Rett individuals).^47^, ^48^ Several studies showed that epilepsy is the major contributor for neuromotor regression in MDS individuals.^47^, ^49^ We also identified epilepsy as the major contributor to parental burden in the caregivers of individuals with MDS.^50^ Age of neuromotor regression and epilepsy onset, and improvement in epilepsy severity can be used as long‐term outcome measures (delaying the age of onset or decreasing the severity) for future interventional trials.

### Neurophysiological studies

Children with MDS and MRL syndromes are known to have seizures and sleep abnormalities, although only very few studies have been collected. More extensive work has been done on RTT.

In RTT, the EEG abnormalities during awake and asleep progress through the four clinical stages. During clinical stage 1 (6–18 months), EEG characteristics typically tend to be normal. Some cases show slowing of the posterior background rhythm during wakefulness.^51^, ^52^ Then, in clinical stage 2 (18 months‐3 years), slow background activity and low occipital‐dominant alpha rhythms is characteristically seen during wakefulness. During NREM sleep, Rolandic spikes (focal spikes in the central and temporal regions) will be observed.^53^ Absent sleep spindles during NREM stage might also be observed.^52^ In clinical stage 3 (2–10 years), sleep patterns continue to be abnormal with low or absent occipital‐dominant alpha rhythm, slow background activity, multifocal spike, and sharp‐wave discharges during wake, absent vertex transients, and absent to few sleep spindles during NREM, and occasional focal spike or sharp‐wave discharges during REM.^52^, ^54^ In the last clinical stage (>10 years), the main abnormalities are similar to stage 3. However, some individuals in this stage may have near‐normal activity with mild slowing of the background activity, a normal occipital‐dominant rhythm during wake, and appearance of spindles during NREM.^52^, ^55^ Some studies of RTT also reported abnormalities spanning multiple clinical stages, including a decrease in N2, an increase of N3, and a decrease of REM sleep.^56^, ^57^

Abnormal EEG findings have been reported in MDS and MRL individuals but with very small subject numbers, and thus have not been comprehensively studied.^58^, ^59^, ^60^, ^61^, ^62^ In those studies, the main abnormal EEG pattern was irregular, slow background activity in wakefulness, and focal spikes during both wake and sleep, which is similar to RTT. However, one study also reported abnormal 12‐Hz, high voltage, asynchronous spindles during sleep in five MDS individuals, which has not been observed in RTT.^58^ In addition, there is only one case series on PSG studies in MDS,^63^ which mainly focused on sleep apnea but did not investigate the sleep structure changes. Given the heritability of many sleep metrics (slow wave sleep power and some spindle parameters), sleep‐EEG is an untapped area of biomarker discovery for genetic syndromes.

We observed several significant differences in sleep physiology between MDS and TD populations. The main differences were as follows: (1) reduced sleeping time, particularly for REM; (2) increased delta power during REM; (3) decreased occipital alpha and increased brain‐wide delta power during wakefulness; and (4) reduced spindle density and duration.

REM sleep is thought to be important for the formation and consolidation of certain types of memory, facilitating cortical plasticity and improving cognitive function, for example, heightening creativity.^64^, ^65^ Therefore, the reduced time in REM sleep, together with the increased delta power during REM, suggest that REM‐related brain functions may be severely disrupted in MDS. High alpha power in the occipital lobe during closed‐eyed wakefulness is thought to be important for visual system function.^40^ Therefore, the reduced occipital alpha power that we observed in MDS may suggest impaired visual processing. One limitation is that the MDS individuals may have kept their eyes open for more of the night. Future study should disambiguate these possibilities. The increased delta power during wakefulness in MDS across the central, frontal, and temporal regions suggests disrupted neural circuit function. A similar increase in wake delta has been observed in RTT^52^ and Angelman syndrome.^66^ It is not yet clear if common, or disease‐specific, developmental mechanisms are responsible for the awake delta rhythmogenesis seen in these NDDs.

Our MRL subjects had lower delta power during NREM 2 and 3 stages than the TD group (Fig. 3H). Some studies have reported that individuals who are affected by Asperger syndrome, respiratory failure, chronic fatigue, and post‐traumatic stress disorder also have lower delta power during NREM stages.^67^ Delta activity during NREM is considered a biomarker of homeostatic sleep drive and is often associated with sleep duration and intensity.^67^ The reduction in delta power during NREM stages might indicate that MRL individuals had worse sleep quality. We saw decreased alpha power and increased delta activity in MDS compared to TD. The decreased power in alpha in MDS might be related to the reduction of REM stage occupancy during sleep. The increased delta power in REM we observed in MDS has been reported in borderline personality disorder.^68^

Sleep spindles are brief bursts of activity in the frequency range of 12 to 15 Hz originating in the thalamus, which occur during NREM sleep.^69^ Spindles are believed to play an important functional role in sleep‐dependent synaptic plasticity and memory consolidation.^70^, ^71^ Altered spindle characteristics, such as reduced spindle density or shorter spindle duration, have been reported in a variety of neurodevelopment disorders, including autism spectrum disorder and RTT patients.^55^, ^72^ In this study, we found alterations of spindle characteristics in both MDS and MRL individuals compared to TD. Similar to RTT, both the MDS and MRL individuals have lower spindle density and altered spindle morphology compared to the TD group, across brain regions (Fig. 2). We also found that there was a difference in spindle duration in the temporal lobes and spindle amplitude in the frontal lobes between MDS and MRL.

The limitations of the study included the following: (1) the small number of participants which may impact results that showed significant variability, preventing stronger conclusions; (2) this study was conducted during the COVID pandemic, thus some of the studies were not conducted longitudinally and not all studies were conducted in both visits; (3) longitudinal assessment was limited to two visits which, preventing a more accurate natural history of the diseases; (4) although we tried to enroll different age groups, matched between cohorts, the selection we were able to obtain could cause bias which may have impacted the results of the study; (5) each patient had different pathogenic variants and two subjects had mosaicism which may result in inter‐individuals variations for all studies; and (6) we included female individuals in the TD cohort, in order to increase statistical power, which may pose gender‐related discrepancies in the neurophysiological studies.

In conclusion, we conducted a prospective, non‐interventional, clinical and imaging/neurophysiological study to determine the differentiating features in *MECP2*‐related disorders. Our studies identified several differences that can be used for safe target engagement and clinical features that can be used to measure improvement in clinical presentation. We also identified that OSA is a previously unnoticed morbidity in both MDS and MRL and all individuals should undergo a sleep study to be screened for OSA.

## Author Contributions

Conceptualization: DP, CC, MM‐S, LM, DGG, CSL, LS, HYZ, MJM, BS. Data curation: DP, CH, HKH, AM. Writing: DP, CH, HKH. Data analysis: CH, HKH, MM‐S, CSL, MJM, BS. Statistics: CH, SA. Supervision and manuscript review: CC, MM‐S, LM, DGG, CSL, LS, HYZ, MJM, BS.

## Funding information

The reported research has been funded by Ionis Pharmaceuticals, Inc. (B.S.), Rett Syndrome Research Trust (RSRT), International Rett Syndrome Foundation (#3701‐1), Doris Duke Charitable Foundation (#2023‐0235), and NINDS K23 NS125126‐01A1 (D.P.), Blue Bird Circle Foundation (B.S., D.G.G.), and the Eunice Kennedy Shriver National Institute of Child Health & Human Development of the National Institutes of Health P50 HD103555 IDDRC grant for the Signature Project (H.K.H., M.M.S., and M.J.M.). The content is solely the responsibility of the authors and does not necessarily represent the official views of the National Institutes of Health and its institutes. The project described was supported in part by the Clinical Translational Core and Circuit Analysis and Modulation Core at Baylor College of Medicine, which is supported by IDDRC Grant Number P50HD103555 from the Eunice Kennedy Shriver National Institute of Child Health & Human Development. The content is solely the responsibility of the authors and does not necessarily represent the official views of the Eunice Kennedy Shriver National Institute of Child Health & Human Development or the National Institutes of Health.

## Conflict of Interest

D.P. and M.J.M. provide consulting service for Ionis Pharmaceuticals, Inc. C.C., L.M., and L.S. are employees of and own stock in Ionis Pharmaceuticals, Inc. Other authors declare no conflict of interest related to this work.

## Supporting information


Figure S1.

Figure S2.

Tables S1‐S8.

Data S1.

## Data Availability

These data are available based with investigators' direct communication to principal investigators of the manuscript.
