# Supplementary figures and images for: Comprehensive assessment reveals numerous clinical and neurophysiological differences between MECP2 ‐allelic disorders
Source: Ann Clin Transl Neurol. 2025 Jan 21;12(2):433–47. doi: 10.1002/acn3.52269 (PMC11822789; doi:10.1002/acn3.52269)

**A**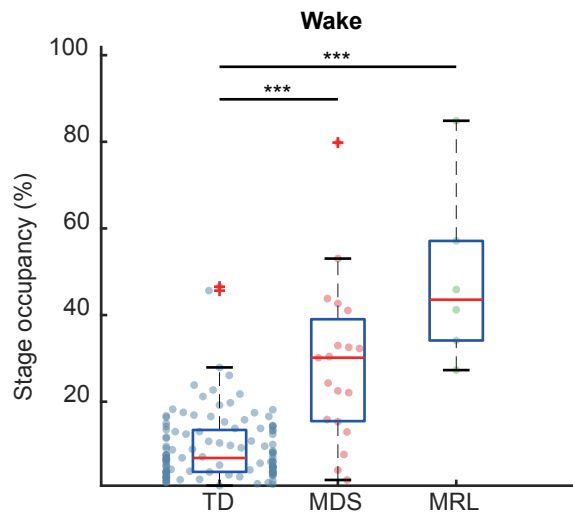**B**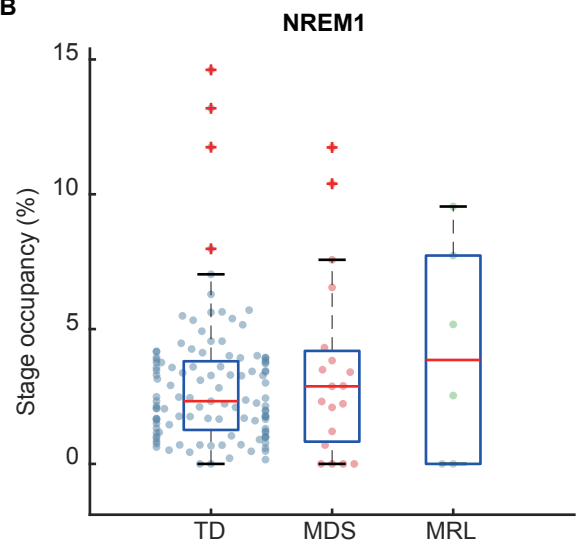**C**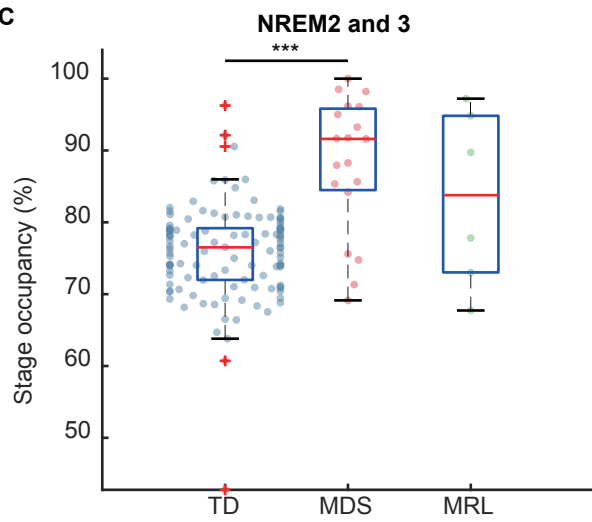**D**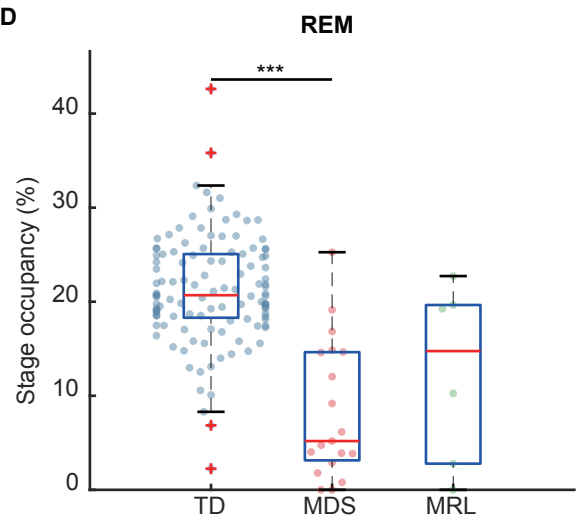

Supplement: Supplementary file 1 — Figure S1. [file ACN3-12-433-s001.pdf]

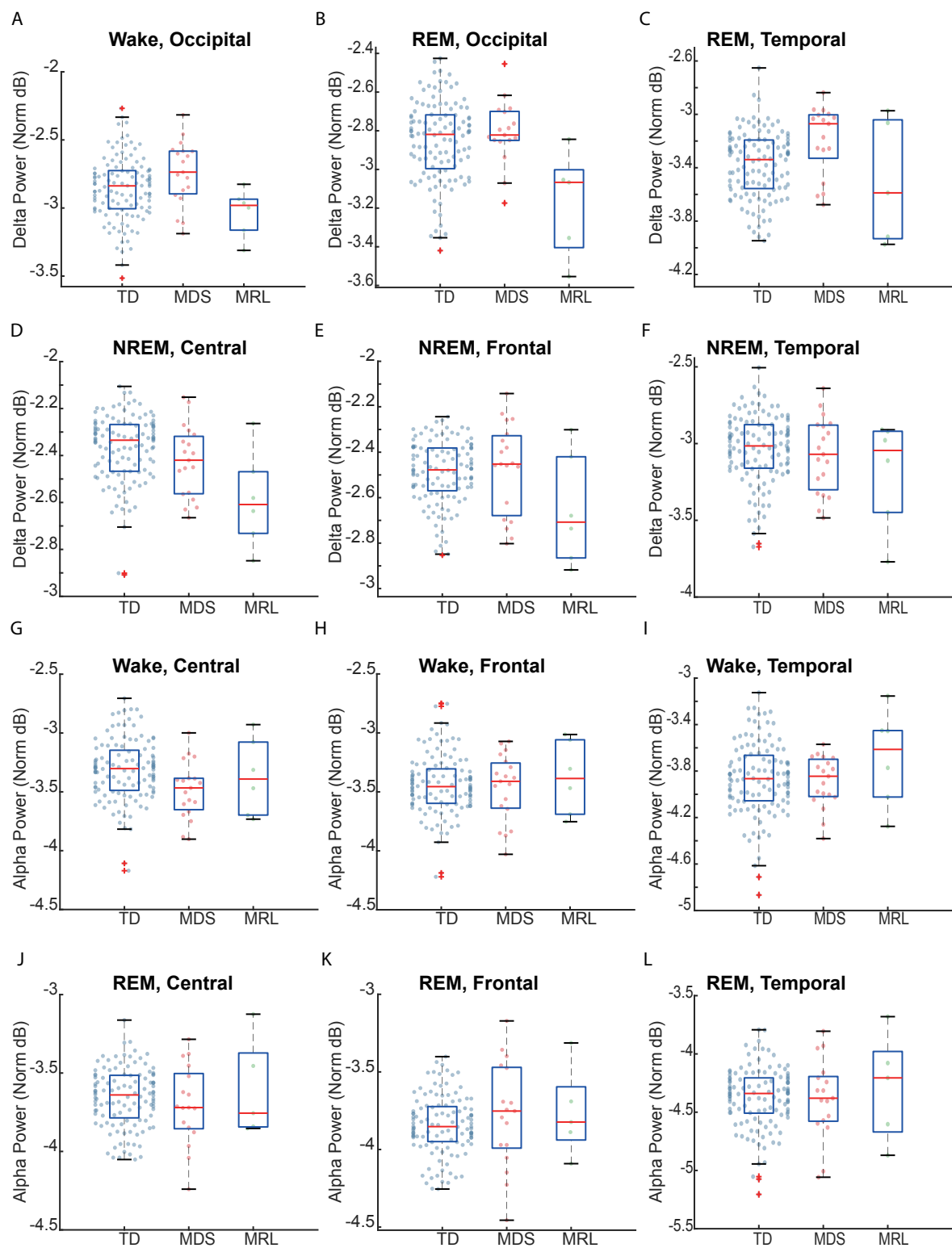

Supplement: Supplementary file 2 — Figure S2. [file ACN3-12-433-s003.pdf]
