# Supplementary material for: Comprehensive assessment reveals numerous clinical and neurophysiological differences between MECP2 ‐allelic disorders
Source: Ann Clin Transl Neurol. 2025 Jan 21;12(2):433–47. doi: 10.1002/acn3.52269 (PMC11822789; doi:10.1002/acn3.52269)
Supplement: Supplementary file 3 — Tables S1‐S8. [file ACN3-12-433-s004.docx]

**Table S1. Molecular details of participants**

| **Subject ID** | **Genomic Information** | | | |
| --- | --- | --- | --- | --- |
|  | **Mutation coordinates (hg19)** | **Transcript reference number** | **Inheritance status** | **Mosaic status** |
| **MDS1** | ChrX:152620676-153836222 | N/A | Inherited | non-mosaic |
| **MDS2** | ChrX:152856816-153536586 | N/A | Inherited | non-mosaic |
| **MDS3** | ChrX:144057799-155251054 (terminal duplication) | N/A | Inherited | non-mosaic |
| **MDS4** | ChrX:153158387-153565901 | N/A | unknown | non-mosaic |
| **MDS5** | ChrX:152814298-153414342 | N/A | Inherited | non-mosaic |
| **MDS6** | ChrX:147326287-155251054 (translocation to chromosome Y) | N/A | Inherited | non-mosaic |
| **MDS7** | ChrX:153065085-153420198 | N/A | Inherited | non-mosaic |
| **MDS8** | ChrX:153073618-153613200 | N/A | Inherited | non-mosaic |
| **MDS9** | ChrX:153108170-153654046 | N/A | Inherited | non-mosaic |
| **MDS10** | ChrX:153246671-153611490 | N/A | Inherited | non-mosaic |
| **MDS11** | ChrX:153206000-153623000 | N/A | Inherited | non-mosaic |
| **MRL1** | c.808delC;p.Arg270fs | NM_004992.4 | de novo | non-mosaic |
| **MRL2** | c.397C>T;p.Arg133Cys | NM_004992.4 | de novo | mosaic |
| **MRL3** | c.401C>G;p.Ser134Cys | NM_004992.3 | de novo | non-mosaic |
| **MRL4** | c.175dupG;p.p.Ala59GlyfsTer32 | NM_004992.4 | de novo | mosaic |
| **MRL5** | c.876-880delTATCC;p.Arg294Cysfs*35 | NM_004992.4 | de novo | non-mosaic |
| **MRL6** | c.48C>T;p.G16= | NM_001110792.2 | de novo | non-mosaic |

ID: Identifier, MDS: MECP2 Duplication Syndrome, MRL: Male Rett-Like Syndrome, N/A: Not applicable

**Table S2. Number of visits per assessment**

|  | **MECP2 Duplication Syndrome** | | **Male Rett Like Syndrome** | |
| --- | --- | --- | --- | --- |
| **Total # of enrolees** | **11** | | **6** | |
| **# of visit status** | **# of single visit** | **# of two visits** | **# of single visit** | **# of two visits** |
| **Comprehensive clinical evaluation** | 3 | 8 | 1 | 5 |
| **Neurodevelopmental assessment** | 7 | 2 | 0 | 5 |
| **Gait** | 2 | 2 | 0 | 3 |
| **Actigraphy** | 3 | 8 | 1 | 5 |
| **Infrared thermography** | 10 | 0 | 6 | 0 |
| **Polysomnography** | 5 | 6 | 1 | 5 |
| **Visual Evoked Potential** | 7 | 1 | 1 | 4 |

**Table S3: Diagnostic metrics of neurophysiological studies**

| **METRICS** | **Formula for MDS (Cut off = 13.5)** | **Formula for MRL (Cut off = 21.1)** | **Awake for MRL (Cut off = 26.65)** |
| --- | --- | --- | --- |
| **Sensitivity (True Positive Rate)** | 0.929 | 1.000 | 0.750 |
| **Specificity (True Negative Rate)** | 0.914 | 0.966 | 1.000 |
| **Positive Predictive Value (PPV)** | 0.565 | 0.429 | 1.000 |
| **Negative Predictive Value (NPV)** | 0.991 | 1.000 | 0.983 |
| **Accuracy** | 0.915 | 0.966 | 0.984 |
| **AUC (Area Under Curve)** | 0.914 | 0.971 | 0.991 |
| ***p*-value** | < 0.001 | < 0.001 | < 0.001 |

MDS: MECP2 Duplication Syndrome, MRL: Male Rett Like

**Table S4. Gait variables - mean and (one standard deviation) separated by participant group.**

| **Variable** | **MDS** | **MRL** | **% Delta** |  | **MDS** | **MRL** | **% Delta** |
| --- | --- | --- | --- | --- | --- | --- | --- |
|  | Left | Left |  |  | Right | Right |  |
| Velocity (m/sec) | 67 (40) | 40 (20) | 68 |  |  |  |  |
| Stride length (cm) | 78 (44) | 66 (25) | 18 |  | 78 (44) | 65 (25) | 20 |
| Step Length (cm) | 39 (20 | 30 (14) | 31 |  | 38 (23) | 35 (12) | 11 |
| Stride time (sec) | 1.3 (0.4) | 1.8 (.56) | -27 |  | 1.2 (0.4) | 1.8 (0.5) | -31 |
| Swing Time (sec) | 0.43 (0.7) | 0.52 (0.20 | -17 |  | 0.36 (0.08) | 0.56 (0.20) | -34 |
| Stance Time (sec) | 0.84 (0.36) | 1.3 (0.67) | -35 |  | 0.87 (0.33) | 1.24 (0.56) | -30 |
| Swing % of gait cycle | 35 (5.4) | 30 (13.3) | 18 |  | 30.5 (7.4) | 32.6 (11.5) | -6.5 |
| Stance % of gait cycle | 65 (5.4) | 70 (13.3) | -7.6 |  | 69.5 (7.4) | 67.4 (11.5) | 3.3 |
| Single Support (sec) | 0.37 (0.1) | 0.56 (0.2) | -34 |  | 0.43 (0.07) | 0.52 (0.25) | -17 |
| Double Support (sec) | 0.48 (0.4) | 0.87 (0.7) | -45 |  | 0.47 (0.3) | 0.86 (0.6) | -44 |
| Single Supp % cycle | 30 (8) | 33 (12) | -8.3 |  | 36 (5) | 30 (13) | 20 |
| Double Supp % cycle | 35 (9) | 42 (19) | -16 |  | 36 (11) | 43 (18) | -15 |

**Table S5. Symmetry - mean and (one standard deviation) separated by participant group.** With the exception of ‘swing time’, the MDS group displays values that reflect fairly good symmetry between the measures of the left and right leg (**Table 3**). The symmetry for the temporal measures in the MRL group are also fairly good, but the two spatial measures, stride length and step length, are far from symmetrical. This indicates that in MRL participants, the two legs are not functioning in an equal (symmetric) manner that is required for walk in a straight line. This is in contrast to the MDS group where patients walked symmetrically. A value of 1 means perfect symmetry between the left and right measures

| Variable | MDS | MRL | % Delta |
| --- | --- | --- | --- |
| Stride length | 0.95 (0.11) | 1.20 (0.31) | -21 |
| Step length | 0.99 (0.13) | 1.23 (0,23) | -20 |
| Stride time | 0.98 (0.04) | 0.99 (0.01) | -1.5 |
| Swing time | 0.85 (0.13) | 0.97 (0.06) | -12 |
| Stance time | 1.04 (0.04) | 0.91 (0.12) | 14 |

**Table S6. Coefficient of Variation - mean and (one standard deviation) separated by participant group.** The coefficient of variation (CV) reflects the relative variability of a given measure. It is the differences in the CVs between the two groups that most strongly suggest significant functional differences between the MDS and MRL individuals. For reference, if the standard deviation equals the mean, the CV would be 100. Thus, the greater the CV, the greater the standard deviation relative to a given mean. The high values observed suggest that the MRL individuals display high levels of gait variability when compared with the MDS individuals.

| Variable | MDS | MRL | % Delta |  | MDS | MRL | % Delta |
| --- | --- | --- | --- | --- | --- | --- | --- |
|  | Left | Left |  |  | Right | Right |  |
| Velocity (m/sec) | 22 (10) | 22 (15) | -16 |  |  |  |  |
| Stride length (cm) | 16 (2) | 19 (13) | -18 |  | 17 (2) | 22 (15) | -34 |
| Step Length (cm) | 16 (1) | 35 (21) | -54 |  | 22 (12) | 22 (12) | 0 |
| Stride time (sec) | 15 (7) | 30 (20) | -53 |  | 11 (7) | 24 (15) | -52 |
| Swing Time (sec) | 23 (23) | 42 (41) | -47 |  | 21 (22) | 28 (25) | -24 |
| Stance Time (sec) | 13 (14) | 26 (24) | -48 |  | 10 (2) | 24 (20) | -59 |
| Swing % of gait cycle | 18 (12) | 28 (19) | -37 |  | 14 (7) | 25 (14) | -43 |
| Stance % of gait cycle | 9 (5) | 13 (14) | -35 |  | 6 (4) | 12 (7) | -46 |
| Single Support (sec) | 21 (23) | 28 (25) | -24 |  | 23 (23) | 42 (41) | -47 |
| Double Support (sec) | 20 (8) | 49 (29) | -59 |  | 19 (2) | 51 (32) | -63 |
| Single Supp % cycle | 1 (8) | 23 (16) | -28 |  | 17 (10) | 31 (21) | -47 |
| Double Supp % cycle | 15 (7) | 22 (8) | -32 |  | 11 (6) | 25 (21) | -57 |

CV = (standard deviation/mean) x 100

**Table S7: Infrared Thermal Assessment values for MDS and MRL groups.**

|  | **Min.** | **1st Qu.** | **Median** | **Mean** | **3rd Qu.** | **Max.** |
| --- | --- | --- | --- | --- | --- | --- |
| **MDS-RH** | 24.7 | 33.15 | 34.45 | 32.82 | 34.92 | 35.5 |
| **MDS-LH** | 26.6 | 30.15 | 34.35 | 32.43 | 34.8 | 35.6 |
| **MDS-RF** | 23.6 | 30.95 | 31.85 | 31.28 | 34.1 | 34.9 |
| **MDS-LF** | 27.4 | 30.4 | 31.25 | 31.67 | 33.98 | 35.1 |
|  |  |  |  |  |  |  |
| **MRL-RH** | 31.9 | 34.3 | 34.6 | 34.73 | 35.6 | 36.8 |
| **MRL-LH** | 33.2 | 34.3 | 34.7 | 35.04 | 35.6 | 37.4 |
| **MRL-RF** | 25.2 | 25.62 | 29.9 | 30.12 | 34.17 | 35.9 |
| **MRL-LF** | 26.2 | 29 | 31.5 | 31.37 | 34.1 | 35.7 |

Temperatures are presented in Celsius.

MDS: MECP2 Duplication Syndrome, MRL: Male Rett Like; -RH: right-hand, -LH: left-hand, -RF: right-foot, -LF: left-foot, Min: minimum, Q2: 1^st^ Quartile, Q3: 3^rd^ Quartile, Max: Maximum

**Table S8: Visual Evoked Potential Means (one Standard Deviation) for MDS and MRL groups**

| **Measurement** | **MDS (N=8)** | **MRL (N=5)** |
| --- | --- | --- |
| **N1 latency** | 121.4 (48.5) | 115.8 (44.5) |
| **P1 latency** | 174.9 (60.1) | 180.5 (74.4) |
| **N2 latency** | 226.9 (74.7) | 239.7 (91.8) |
| **N1-P1 amplitude** | 3.7 (2.2) | 4.4 (0.9) |
| **P1-N2 amplitude** | 5.2 (3.2) | 4.8 (2.6) |
