# Supplementary material for: Comprehensive assessment reveals numerous clinical and neurophysiological differences between MECP2 ‐allelic disorders
Source: Ann Clin Transl Neurol. 2025 Jan 21;12(2):433–47. doi: 10.1002/acn3.52269 (PMC11822789; doi:10.1002/acn3.52269)
Supplement: Supplementary file 4 — Data S1. [file ACN3-12-433-s002.docx]

**SUPPLEMENTARY NOTE**

**SUPPLEMENTARY MATERIALS and METHODS for Polysomnography (PSG) study**

To clean the PSG data, we removed epochs with an overlapping arousal, movement, or signal artifact annotation. Following this, aberrant epochs with artifacts caused by limb, head or eye movements, or jaw, neck muscle contraction were identified and removed. For each 30-s epoch, we calculated spectral band power for the 1–4 Hz and 15–30 Hz bands by multitaper spectral analysis (14 discrete prolate spheroidal sequences, DPSS).^31^ To remove remaining aberrant epochs, we compared each epoch to the moving average based on up to 15 contiguous epochs (7 on either side of the ‘current’ epoch), and an epoch was excluded if the delta power was >2.5 times the local average, or if the beta power was >2.0 times the local average.^32^

For each subject, we calculated average power in each spectrum band. Specifically, we estimated power spectral density of each pre-processed 30-s epoch signal using multitaper spectral analysis (14 DPSS). To account for uninformative individual differences in signal amplitude, resulting, for example, from skin thickness and surface conductivity, and retain within-subject topographical patterns and sleep stage differences, we normalized the spectrum power of each electrode by the averaged total power (from 0-30 Hz) across all electrodes and time points for each session, as suggested by Cox et al.^33^ Then, for each session, we calculated the averaged power at the following 6 spectrum bands: delta (2-4 Hz), theta (5-8 Hz), alpha (8-10 Hz), sigma (12-15 Hz), fast sigma (16-18 Hz), and slow beta (18-20 Hz), for each stage. All normalized power values are base-10 log transformed before plotting and statistics.

Trained sleep experts from Texas Children’s Hospital Sleep Lab scored each 30 second epoch into 5 stages (wake, rapid eye movement (REM), and non-REM 1-3) based on the EEG recordings in accordance with American Association of Sleep Medicine (AASM) criteria.^34^ However, for some MDS and MRL individuals with highly abnormal EEG patterns, it was difficult for the technician to differentiate between NREM2 and 3. Therefore, for consistency we grouped stages NREM2 and 3 together for both control and patient groups in all analyses.

Spindles were detected from NREM stages (NREM2 and 3) signals across all electrodes using the Luna spindle detector, which is an open-source software package for manipulating and analyzing polysomnographic recordings, with a focus on the sleep EEG.^32^ The spindle detector is based on the Morlet wavelet transformation. Briefly, we extracted the wavelet coefficients of the EEG signals at the center frequency of 13 Hz. The wavelet coefficients of the sample points were smoothed using a moving average (window duration of 0.1 s). Sample points with coefficients greater than a multiplicative threshold (4.5 times greater than the average value across all artefact-free NREM2 and 3 sleep for that individual/channel) were flagged. Intervals of consecutively flagged points with durations between 0.3 and 3.0 s were labelled as putative spindles. We then applied the inclusion criteria described in Purcell et al^32^ to exclude false spindles.

Three spindle characteristics were extracted from these putative spindles: 1) spindle density, the number of spindles per minute across the NREM stages; 2) spindle amplitude, the root mean squared (RMS) of the spindle signals, normalized by the RMS of the EEG signals during overall sleep; and 3) spindle duration, the duration of each spindle, in seconds.

***Additional Statistical Analyses methods***

We averaged spindle (density, normalized amplitude, and duration) and power spectral metrics for the leads within each brain region. Specifically, leads C1, C2 and Cz were grouped as the central region, leads Fp1, Fp2, F7, F8, and Fz as the frontal region, leads O1, O2 and Oz as the occipital region, and leads T3 and T4 as the temporal region. To assess the difference of these spindle and spectral estimates between the two visits of MDS individuals, we used a Wilcoxon signed rank test. We used a Kruskal-Wallis ANOVA test to assess spindle characteristics and power spectrum differences between the TD, MDS, and MRL groups at different regions, followed by Tukey’s post-hoc test for correction. As above, repeat visits of MDS patients were included as independent data points. To account for the number of spectral bands being compared, we further multiplied the p-value by 6 (a Bonferroni-type correction), and the significance threshold was set as p < 0.05 after the correction.

**SUPPLEMENTARY RESULTS**

**Comprehensive Clinical Evaluation:**

There were no differences in the frequency of gastrointestinal problems, poor eye contact, constipation, chewing and swallowing difficulties, G-tube feeding, gastroesophageal reflux, dysautonomia, insomnia, obstructive sleep apnea (OSA), genitourinary abnormalities, bruxism, high pain tolerance, and self- and others-mutilation (**Table 1**)

While there were no differences in the frequency of dysautonomia symptoms, MDS individuals had relatively mild symptoms such as drooling, and color and temperature change in the extremities while MRL individuals additionally had classic RTT breathing abnormalities (both breath-holding and/or hyperventilation). OSA was globally present in both groups thus did not have a statistical difference. Similar to dysautonomia, MRL individuals were found to have more severe OSA compared to MDS individuals. Importantly, 6 out of 8 MDS individuals were found to have OSA as assessed during the study visits. Behaviorally, MDS individuals were either overactive (4/11) or overly passive (2/11), while four out of six MRL individuals were overly passive.

Poor eye contact, abnormal activity (MDS individuals are more active while MRL individuals are overly passive), and bruxism are other behavioral findings common in both MDS and MRL. We also found that high pain tolerance is also universal both in MDS and MRL individuals.

Longitudinally (two visits 8-10 months apart), young MDS individuals (<6 years) continued to gain new skills. One 12-year-old MDS individual showed severe worsening of epilepsy and MDS individuals >15-year-old were relatively stable. MRL individuals had scattered clinical presentations on the follow up visit with no correlation between individuals’ age, gaining new skills or regression.

***Neurodevelopmental Assessment***

Neurodevelopmental assessments were performed on 7 MDS and 5 MRL individuals during Visit #1, and repeated on 2 MDS and 5 MRL individuals during Visit #2 which occurred 8-10 months after visit #1 (**Table S2**). Gross motor functioning on direct assessment was similar between MDS and MRL individuals with skills ranging from 4 months to 12 months age equivalents for both cohorts. Via parent report of gross motor skills on the Vineland-3, functioning ranged from 4 months up to 21 months.

Fine motor/visual perceptual/problem solving skills assessment with the Capute Scales, MDS individuals displayed skills ranging from 6 months up to 21 months of age while MRL individuals’ highest skill attainment was at 10.4 months (range 4 months to 12 months). Parents reported similar fine motor skill ranges on the Vineland-3.

Expressive language skills via direct assessment ranged from 8–11 months in MDS individuals and 4–9 months in MRL individuals. Receptive language skills ranged from 4–18 months for MDS individuals and 4–10 months for MRLs. Parent report on the Vineland-3 was consistent for both language skills.

Longitudinal data was available from 2 MDS individuals and 5 MRL individuals. In one younger MDS individual, there were small improvements on Capute scales scores, while the other MDS individual, an older individual who experienced medical complications in the interim, demonstrated a small decrease in scores. In MRL individuals, the pattern was mixed with 3 showing small increases in scores while 2 individuals had an increase in only one of the two Capute Scales domains. One individual’s scores did not change.

Results suggest that MDS individuals may have a higher level of maximum skill attainment (higher age equivalent reached) than MRL individuals in certain skills (fine motor/visual motor problem solving and receptive language), but small sample size limits any firm conclusions. Limitations to developmental assessments include variability in child attention and performance, environmental factors as noted above (some individuals receive intensive services while others do not), and the age of individuals at a cross-sectional time point, in light of knowledge of natural history of disease (regression, onset of seizures, etc.). Many individuals’ parents reported a history of regression of skills, particularly in the MRL cohort, and thus a child’s highest level of skill attained in the past could have been higher than that measured on direct observation at the time of assessment. In those individuals whom we were able to assess longitudinally, developmental tests showed consistency from both direct observation measures and standardized parent report of functioning measures however, data was limited to make powerful conclusions. At the same time, developmental measures were able to capture even small differences between visits. Having multiple points of assessment helps protect against the variability that might be present due to behavioral or environmental factors. The combination of direct assessment and parent report is optimal.

***Gait Assessment***

While gait has been studied extensively in Rett syndrome, it has not been studied in MDS individuals. Therefore, we assessed gait in three ambulatory MDS individuals and compared to three ambulatory MRL individuals. Given the low number of participants in each group and large variability within the gait variables, it was appropriate to present descriptive statistics to represent the emerging trends within the data.

The average number of steps obtained for each individual was 87 (SD = 18), ranging from 58 to 107 steps (**Table S4**). MDS group tended to demonstrate less variability, particularly of the temporal variables as well as a clear trend to have lower values in the almost all of these variables compared to the MRL group. Except for the two spatial variables (i.e., step and stride length) of the MRL group, symmetry measures reflected that the two limbs were moving in a similar manner (**Table S5**). The coefficients of variability, displayed in **Table S6**, indicate that the relative variability of almost all of the measures of the MDS group trend toward being less than the MRL group.

Although the gait study was conducted in a small subset of individuals, it demonstrated differences between MDS and MRL with MRL individuals displaying high levels of gait variability when compared with the MDS individuals. MDS individuals also had better symmetry and MRL individuals required more directional correction. Gait is relatively well studied in RTT,^1-3^ however there have been no prior gait studies conducted on MDS individuals. Gait studies should be repeated with a larger number of MDS individuals to validate the differences we identified in our study, in order to use gait as potential outcome measure or measure of clinical difference between MDS and MRL.

***Actigraphy***

All 17 participants (11 MDS and 6 MRL) completed actigraphy assessments and 13 of them (8 MDS and 5 MRL) completed two assessment sessions (**Table S2**). We compared sleep onset and wake-up times calculated from the PSG studies to the measurements obtained from the actigraphs. Times of the sleep onset and offset identified by actigraph were within 40 minutes of those identified by PSG in both groups, which suggests actigraphy as a reliable measurement tool for assessing sleep vs. wakefulness in MDS and MRL. Next, we examined the effect of tremor on actigraphy measurements. We compared mean values from the individual with the most visible tremor on exam with the whole group and did not find increased activity values. Thus, we conclude that accurate determination of sleep patterns can be achieved with the PhilipsActiwatch in MDS and MRL individuals.

In over half of our participants, we found a consistent pattern of activity across days (more often with higher activity in the mornings). This circadian activity difference was not statistically different between the MDS and MRL groups (p= 0.623).

Although actigraphy was conducted in the entire cohort and most of the participants had it performed during two visits for an extensive duration (7 days), we did not detect a difference in daily activity patterns between MDS and MRL. This can in part be due to the small sample size in our cohort. Although actigraphy does not appear to be a good differentiating marker of MDS vs. MRL, but it may be used as an outcome measure for interventional treatment.

***Infrared Thermal Assessment***

Dysautonomia resulting in cold and discolored extremities is one of the hallmarks of RTT, however there are no studies assessing dysautonomia in MDS. Therefore, we used infrared thermal assessment to measure skin temperature. Data were successfully obtained from 2 MDS individuals and 3 MRL individuals in the first round of patient visits, and from 8 MDS and 5 MRL individuals in the second set of visits (**Table S2**). In contrast to findings in girls with RTT^4^, skin temperature measurements were not significantly different between body parts and no significant asymmetry of measurements was found (**Table S7**). Comparing right hand versus left hand, right foot versus left foot, or between hands and feet, did not yield significant differences, for neither the MDS nor the MRL group (left versus right MDS hands p=1.0 and feet 0.94, MRL p=0.87 and 0.63; all hands MDS vs MRL p=0.16 and all feet p=0.74). Also, comparing measurements between the MDS and MRL groups, we did not detect significant differences (p= 0.39). Of note, physical examination showed that cold hands/feet were common in both MDS and MRL, and this validates the infrared thermal assessment finding and literature.^5, 6^

Similar to actigraphy, although infrared thermal assessment did not show difference between MDS and MRL, it can be used as an outcome measure for interventional studies.

***Visual Evoked Potential***

Eight MDS individuals and five MRL individuals were studied. One MDS and four MRL individuals underwent two assessments (**Table S2**). The latencies of N1, P1, and N2 were not significantly different between the MDS and the MRL groups (N1: p=0.86 (left eye) and p=0.56 (right eye) and P1: p=0.68 (left eye) and p=0.76 (right eye) and N2: p=0.71(left eye) and p=0.94 (right eye)). However, latency averages for both groups were (much) longer than those reported in LeBlanc for Typically Developing (TD) and RTT (**Table S8**).^7^

Latencies did not significantly differ between both eyes (monocular testing). For those patients where a longitudinal comparison was possible, we found P1 latencies that differed by up to 79ms (142ms and 221ms) between visits, likely demonstrating the variability of participation/focusing on target by some individuals.

N1-P1 and P1-N2 amplitudes were lower than those reported for TD, however, N1-P1 and P1-N2 amplitudes were not significantly different (N1-P1: p=0.41 (left eye) and p=0.49 (right eye); P1-N2: p=0.84 (left eye) and p=0.68 (right eye) between MDS and MRL (**Table S8**)*.*

These results were consistent with what Saby et al. previously reported in Rett and related disorders.^8^ In addition, N1, P1 and N2 latencies were prolonged compared to TDs. While it is known that latencies can be prolonged in neurodevelopmental disorders,^9^ it is feasible that some of the differences in latencies can be attributed to the marked attentional deficits that both the MDS and MRL groups portray.^10, 11^

**Time awake and in sleep stages**

We first evaluated the difference in the time spent in the wake stage for each visit of MDS individuals. The p-value, when comparing the wake percentage between the two visits for each MDS individuals (visit 1: 35.2% ± 6.9%, visit 2: 21.8% ± 5.3%) was 0.055, indicating a marginally significant result. Thus, these visits were not pooled, but rather included as independent data points for further analysis. We then compared time spent awake among the 3 groups (MDS, MRL, TD). A Kruskal-Wallis test showed that there was a significant difference of means at the group level (Chi square = 36.71, p < 0.001). Post-hoc pairwise comparison showed that compared to typical developing (TD), both the MDS and MRL groups spent significantly more time in the awake stage (**Fig. S1A**). No significant difference was found between MDS and MRL groups in time spent awake (p = 0.12).

We then excluded the wake stage and evaluated the percentage of sleep time spent in each of the resolvable sleeping PSG stages (NREM1, NREM2/3, or REM). For stage NREM1, no significant difference was found between the two visits of MDS individuals (visit 1: 3.2% ± 0.9% and visit 2: 3.6% ± 1.5%, p = 0.94). For both the combined NREM2-3 stages, and the REM stage, the p-value when comparing stage percent occupancy during the two visits of MDS individuals (for NREM 2 and 3 stages: visit 1: 91.6% ± 2.4%; visit 2: 84.3% ± 3.7%; for REM stage: visit 1: 5.2% ± 1.7%; visit 2: 12.1% ± 2.7%) was significant (p=0.05). No significant differences of the duration of the NREM1 stage between TD, MDS, and MRL groups were found (p = 0.86) (**Fig. S1B**).

For both NREM2-3 stages and REM stages, a Kruskal-Wallis test showed group-level effects (NREM 2-3, Chi square = 22.42, p < 0.001. REM, Chi square = 36.02, p < 0.001). Subsequent pairwise comparisons showed that the MDS individuals spent significantly more time in the combined NREM2-3 stages than the TD group, but had a similar percentage to the MRL group (**Fig. S1C**). The MDS group also spent much less time in REM stage than the TD group (**Fig. S1D**), but similar to the MRL group (p = 0.58). The time spent in NREM 2-3, and REM sleep stages in MRL group was not altered significantly compared to TD group (NREM 2-3, p = 0.39; REM, p = 0.07). For stage NREM1, no significant difference was found between the two visits of MDS individuals (p = 0.94).

**SUPPLEMENTARY FIGURE LEGENDS**

**Figure S1:** **Altered PSG stage occupancy in MDS and MRL.** **(A)** The percentage of the sleep session spent in the awake state, as indicated by the PSG manual scoring**.** Both the MDS and MRL groups displayed significantly higher time in wake stage than the control (TD) group. **(B)** Stage occupancy for NREM1. No significant difference was found between the percentage of NREM1 stage at the group level. **(C)** Stage occupancy for NREM2 and 3, combined. The percentage time of the combined N2 and N3 stages in the combined MDS group is significantly higher than the TD group (N = 150). **(D)** Stage occupancy for REM. The TD group had a significantly higher percentage of REM stage than the combined MDS group. The red line for each box plot indicates the median, and the bottom and top edges of the indicate the 25th and 75th percentiles, respectively. Comparison of wake duration between groups was performed by the Kruskal-Wallis test followed by Tukey’s post-hoc multiple comparison test. ***p<0.001 reflect post hoc tests. Red ‘+’ indicates values outside the quantile range. Results are from 19 MDS, 6 MRL, and 150 TD visits.

**Figure S2: Brain regions without altered delta and alpha in the indicted PSG stage.** (**A-L**) power in indicated frequency band for indicated brain region and sleep stage in TD, MDS, and MRL. Red ‘+’ indicates values outside the quantile range. Results are from 19 MDS, 6 MRL, and 150 TD visits.

**REFERENCES**

1. Layne CS, Young DR, Lee BC, et al. Kinematics associated with treadmill walking in Rett syndrome. *Disabil Rehabil* 2021; 43: 1585-1593. 2019/10/16. DOI: 10.1080/09638288.2019.1674389.

2. Young DR, Suter B, Levine JT, et al. Characteristic behaviors associated with gait of individuals with Rett syndrome. *Disabil Rehabil* 2022; 44: 1508-1515. 2020/09/16. DOI: 10.1080/09638288.2020.1820084.

3. Layne CS, Lee BC, Young DR, et al. Temporal Gait Measures Associated With Overground and Treadmill Walking in Rett Syndrome. *J Child Neurol* 2018: 883073818780471. 2018/06/22. DOI: 10.1177/0883073818780471.

4. Symons FJ, Byiers B, Hoch J, et al. Infrared Thermal Analysis and Individual Differences in Skin Temperature Asymmetry in Rett Syndrome. *Pediatr Neurol* 2015; 53: 169-172. 2015/05/25. DOI: 10.1016/j.pediatrneurol.2015.03.018.

5. Peters SU, Fu C, Suter B, et al. Characterizing the phenotypic effect of Xq28 duplication size in MECP2 duplication syndrome. *Clin Genet* 2019; 95: 575-581. 2019/02/23. DOI: 10.1111/cge.13521.

6. Miguet M, Faivre L, Amiel J, et al. Further delineation of the MECP2 duplication syndrome phenotype in 59 French male patients, with a particular focus on morphological and neurological features. *J Med Genet* 2018; 55: 359-371. 2018/04/06. DOI: 10.1136/jmedgenet-2017-104956.

7. LeBlanc JJ, DeGregorio G, Centofante E, et al. Visual evoked potentials detect cortical processing deficits in Rett syndrome. *Ann Neurol* 2015; 78: 775-786. 2015/09/04. DOI: 10.1002/ana.24513.

8. Saby JN, Peters SU, Benke TA, et al. Comparison of evoked potentials across four related developmental encephalopathies. *J Neurodev Disord* 2023; 15: 10. 2023/03/05. DOI: 10.1186/s11689-023-09479-9.

9. Kim J, Sung IY, Ko EJ, et al. Visual Evoked Potential in Children With Developmental Disorders: Correlation With Neurodevelopmental Outcomes. *Ann Rehabil Med* 2018; 42: 305-312. 2018/05/17. DOI: 10.5535/arm.2018.42.2.305.

10. Callaway E and Halliday R. The effect of attentional effort on visual evoked potential N1 latency. *Psychiatry Res* 1982; 7: 299-308. 1982/12/01. DOI: 10.1016/0165-1781(82)90066-x.

11. Di Russo F and Spinelli D. Electrophysiological evidence for an early attentional mechanism in visual processing in humans. *Vision Res* 1999; 39: 2975-2985. 2000/02/09. DOI: 10.1016/s0042-6989(99)00031-0.
